# Targeted inhibition of heat shock protein 90 disrupts multiple oncogenic signaling pathways, thus inducing cell cycle arrest and programmed cell death in human urinary bladder cancer cell lines

**DOI:** 10.1186/1475-2867-13-11

**Published:** 2013-02-08

**Authors:** Panagiotis K Karkoulis, Dimitrios J Stravopodis, Eumorphia G Konstantakou, Gerassimos E Voutsinas

**Affiliations:** 1 Laboratory of Environmental Mutagenesis and Carcinogenesis, Institute of Biosciences and Applications, National Center for Scientific Research (NCSR) “Demokritos”, Aghia Paraskevi, 15310, Athens, Greece; 2 Department of Cell Biology and Biophysics, Faculty of Biology, University of Athens, Panepistimiopolis, Zografou, 15784, Athens, Greece

**Keywords:** Bladder, Cancer, Hsp90, Geldanamycin, Signaling

## Abstract

**Background:**

Geldanamycin (GA) can be considered a relatively new component with a promising mode of action against human malignancies. It specifically targets heat shock protein 90 (Hsp90) and interferes with its function as a molecular chaperone.

**Methods:**

In this study, we have investigated the effects of geldanamycin on the regulation of Hsp90-dependent oncogenic signaling pathways directly implicated in cell cycle progression, survival and motility of human urinary bladder cancer cells. In order to assess the biological outcome of Hsp90 inhibition on RT4 (grade I) and T24 (grade III) human urinary bladder cancer cell lines, we applied MTT assay, FACS analysis, Western blotting, semi-quantitative (sq) RT-PCR, electrophoretic mobility shift assay (EMSA), immunofluorescence and scratch-wound assay.

**Results:**

We have herein demonstrated that, upon geldanamycin treatment, bladder cancer cells are prominently arrested in the G1 phase of cell cycle and eventually undergo programmed cell death via combined activation of apoptosis and autophagy. Furthermore, geldanamycin administration proved to induce prominent downregulation of several Hsp90 protein clients and downstream effectors, such as membrane receptors (IGF-IR and c-Met), protein kinases (Akt, IKKα, IKKβ and Erk1/2) and transcription factors (FOXOs and NF-κΒ), therefore resulting in the impairment of proliferative -oncogenic- signaling and reduction of cell motility.

**Conclusions:**

*In toto*, we have evinced the dose-dependent and cell line-specific actions of geldanamycin on cell cycle progression, survival and motility of human bladder cancer cells, due to downregulation of critical Hsp90 clients and subsequent disruption of signaling -oncogenic- integrity.

## Background

Heat shock protein 90 (Hsp90) is an abundant intracellular protein that has emerged as a significant target of modern cancer therapeutic protocols. Hsp90 normally accounts for ~1-2% of the total protein content of the cell, while under stress conditions its levels increase up to ~4-6% of the whole cytosolic proteomic load [1-3]. Hsp90 chaperone activity depends on its transient dimerization, which stimulates the essential, intrinsic ATPase function. The Hsp90 chaperone complex maintains the correct conformational folding, functional integrity and proteolytic turnover of a broad range of protein clients that are implicated in various signal transduction pathways controlling, among others, cell survival and proliferation [2,4]. Moreover, a number of oncogenic proteins critically depend on the Hsp90 molecular chaperone to obtain an active conformation and a functional capacity. Hsp90 upregulation in several malignancies and solid tumors unveils a likely “protective” role of the chaperone in tumorigenesis, serving as a “biochemical buffer” [5]. Hsp90 represents an indispensable part of a complicated machinery called “chaperosome” that allows cancer cells to escape normal regulation and function. Hence, cancer initiation, tumor progression and its clonal evolution are associated with a requirement for increased intracellular levels of Hsp90 [6-8]. Therefore, due to cancer cell dependence upon specific -oncogenic- Hsp90 protein clients, Hsp90 inhibition is thought to negatively interfere with critical oncogenic signaling pathways involved in the hallmark traits of cancer (i.e. sustaining cell proliferation, resisting cell death, and promoting invasion and metastasis) [9], demonstrating exciting prospects in the future of cancer therapeutics [10].

Urinary bladder cancer ranks second in frequency among malignancies of the genitourinary tract and is the fifth most commonly diagnosed non-cutaneous neoplasm of the industrialized world, accounting for more than 4% of all types of cancer worldwide [11-13]. A significant percentage of bladder cancer patients (~70-80%) initially present with highly differentiated, non-invasive papillary tumors, whereas the rest (~20-30%) develop an aggressive muscle-invasive tumor of low differentiation. Although the vast majority of patients are diagnosed with superficial tumors, ~30% of the initial cases are estimated to recur to invasive forms within a time-span of two years. On the other hand, more than ~50% of the patients with primarily invasive tumors develop metastases over a time-period of two years, while the five-year survival rate for metastatic disease is calculated as low as ~5% [14]. Human urothelial tumors are known to evolve along two major and independent biological axes, each presenting with diverse and discrete genetic alterations controlling tumor initiation and progression [15,16]. The complexity of the molecular pathways involved in bladder cancer onset, combined with the various genetic and epigenetic events that occur during tumor progression, are mainly responsible for the great heterogeneity of the disease [17,18]. Established systemic chemotherapy regimens -with conventional drugs- or bladder irradiation have not presented with strong potency against superficial and metastatic urothelial carcinomas, while new clinical protocols designed for targeted therapy are currently tested as second generation treatment-strategy [19].

In this context, we have, herein, examined the effects of geldanamycin, a prototype member and “parent” compound of a group of antibiotics widely known as benzoquinone ansamycins (BAs), on human tumorigenic urothelium. Geldanamycin proved the first natural product carrying Hsp90 inhibitory properties that was able to critically interfere with a complex network of oncogenic signaling pathways regulating survival, proliferation and motility of the two -representative for the disease- human urinary bladder cancer cell lines, of diverse malignancy grade and *p53* genetic content, RT4 (grade I; wild-type *p53*) and T24 (grade III; mutant *p53*). In terms of morphology, genetic content, response to stress, tumorigenicity and metastatic potential, RT4 and T24 cell lines reliably reflect the *in vivo* cellular environments of low and high grade bladder malignancies, respectively, in affected human patients.

## Results and discussion

### Geldanamycin inhibits cell cycle progression of human urinary bladder cancer cells

**Figure 1:**
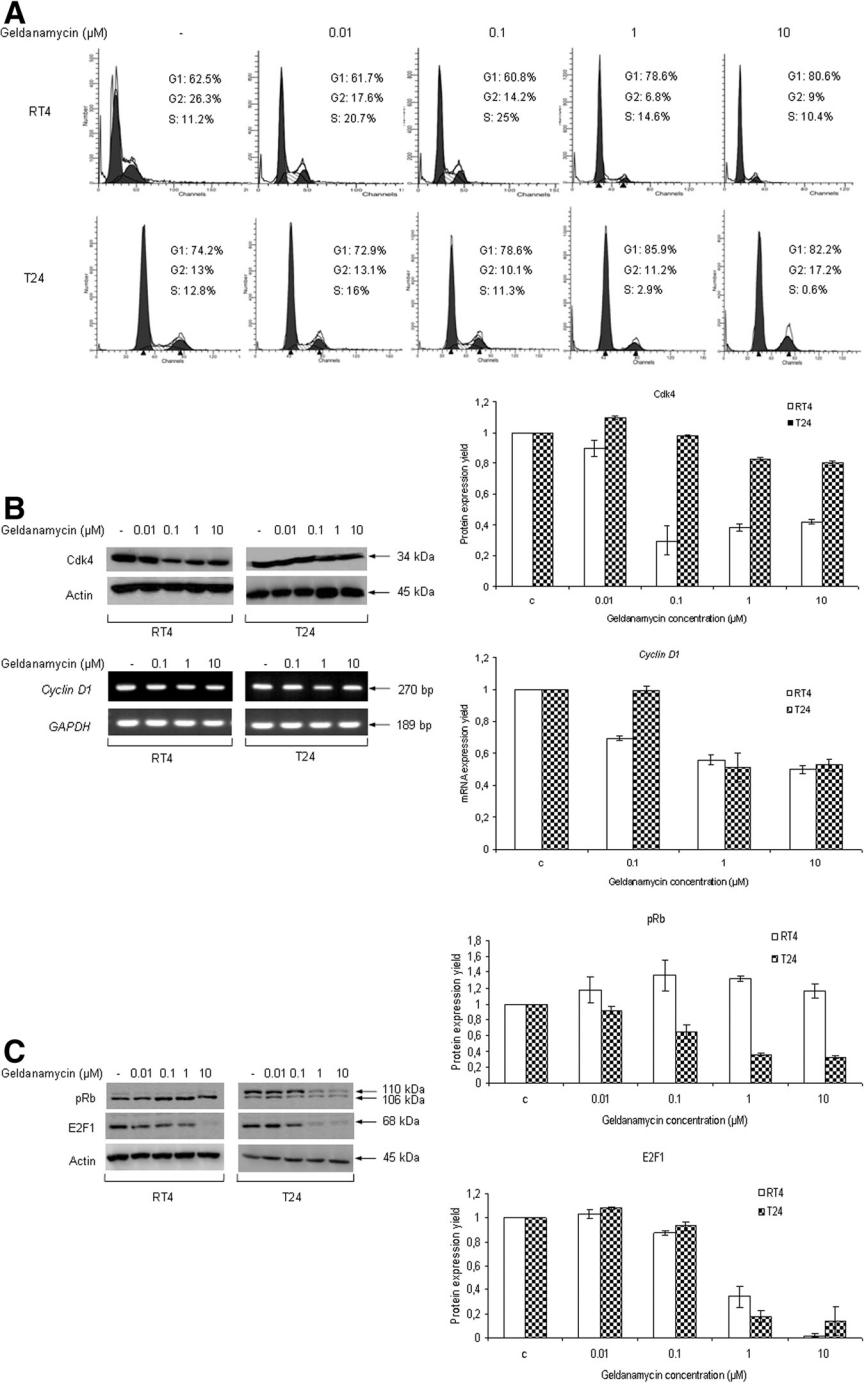
**Reduction of bladder cancer cell cycle activity in response to geldanamycin.** (**A**) Representative FACS analysis of RT4 and T24 human urinary bladder cancer cells. Total cell sub-population percentages at each cell cycle phase are shown. FACS experiments were repeated three times. (**B**) Detection of protein (Cdk4) and mRNA expression (*Cyclin D1*) levels of the Cyclin/Cdk complex -critical- components responsible for the G1→S transition of cell cycle in RT4 and T24 cells. Left panel: images of protein (top) and RNA transcript (bottom) expression profiles after geldanamycin administration. Right panel: protein (top) and RNA transcript (bottom) densitometric quantification bars, denoting the drug-induced alterations of Cdk4 and *Cyclin D1* expression levels compared to control conditions, using Actin (upper left panel) and *GAPDH* (lower left panel) as protein and gene of reference, respectively. (**C**) pRb and E2F1 protein expression profiles in response to 24 hours of geldanamycin treatment in RT4 and T24 bladder cancer cells. Left panel: images of protein expression profiles after geldanamycin administration. Right panel: protein densitometric quantification bars, denoting the drug-induced alterations of pRb (top) and E2F1 (bottom) expression levels compared to control conditions, using Actin (left panel) as protein of reference. Western blotting and sqRT-PCR experiments were carried out three times, one of which is respectively shown here. Standard deviation values are depicted as error bars on top of each value.

We have studied the effect of 24-hour geldanamycin treatment on the progression of the cell cycle of RT4 and T24 human urinary bladder cancer cells by the use of flow cytometry (Figure 1A). RT4 presented with a dose-dependent G1 arrest (from 62.5% in the control to 80.6% at 10 μM), while T24 cells displayed a similar pattern of cytostatic effect, with the percentage of G1-trapped cells rising to 85.9% (from 74.2% in the control) at the concentration of 1 μM geldanamycin. T24 cells also proved to obtain a mild G2-block (13% in the control to 17.2% at the dose of 10 μΜ). In order to mechanistically illuminate the G1-arrest observed, we examined the effect of geldanamycin on several modulators of the cell cycle, such as Cyclin/Cyclin dependent kinase (Cdk) complex proteins (Figure 1B) and downstream cell progression effectors, such as pRb (retinoblastoma protein) and the transcription factor E2F1 (Figure 1C). As shown in Figure 1B (upper panel; image and graph), Cdk4 protein levels follow a cell line-specific response to increasing concentrations of geldanamycin, with RT4 exhibiting a dose-dependent decrease of Cdk4 expression levels up to the dose of 0.1 μΜ that is followed by a subsequent rise of protein levels at the highest concentrations (1 and 10 μM), therefore disrupting the downregulation pattern. In contrast, the highly malignant T24 cells presented with a moderate and dose-dependent downregulation profile of Cdk4 levels in response to the drug. The study of the expression levels of *Cyclin D1* mRNA revealed a similar pattern of dose-dependent downregulation in both drug-treated cell lines (Figure 1B {lower panel; image and graph}), likely indicating the Cyclin/Cdk complex implication in the geldanamycin-induced G1 cell cycle arrest. As shown in Figure 1C (image and graphs), the expression levels and activity status of the critical cell cycle regulators pRb and E2F1 were also analyzed through Western blotting. RT4 presented with a slight increase of -total- pRb protein levels up to the concentration of 1 μM geldanamycin, whereas T24 cells exhibited a notable reduction of its expression (the lower band of 106 kDa) in a dose-dependent manner. The differentiated RT4 cells do not carry any (multi-)phosphorylated pRb form(s), while malignant T24 cells are characterized by the presence of a constitutively (multi-)phosphorylated pRb protein form (the upper band of 110 kDa) [20], which follows a dose-dependent downregulation in response to geldanamycin exposure. Furthermore, E2F1 protein expression levels displayed a prominent downregulation in both drug-treated cell lines, rendering the transcription factor almost undetectable at the higher dose of 10 μΜ and therefore suggesting its critical implication in the geldanamycin-induced G1-block described here. Hsp90 inhibition is known to facilitate cell cycle arrest in all checkpoints of the cell cycle, depending on malignancy grade and cellular context [21]. In the bladder cancer cell lines examined in the present study, geldanamycin administration primarily leads to a dose-dependent G1-checkpoint cell cycle arrest, while analysis of expression and activation profiles of several determinants of the cell cycle (Cdk4, pRb, Cyclin D1 and E2F1) correlate well with the observed block in cycle progression. Furthermore, the geldanamycin-induced E2F1 strong downregulation profile demonstrated herein is a novel and interesting finding. The molecular mechanism underlying E2F1 protein downregulation upon Hsp90 inhibition (i.e. transcriptional suppression, ubiquitination and proteasomal degradation, and autophagic cell death) is still elusive and remains to be further explored. To conclude, Hsp90 inhibition due to geldanamycin administration proved to induce a severe reduction in the expression dynamics of several *bona fide* Hsp90 protein clients involved in cell cycle progression (concurring with chaperones’ ability to regulate cell cycle [22]), subsequently diminishing bladder cancer cell proliferation potential.

### Geldanamycin manifests a cytotoxic effect on human bladder cancer cells

**Figure 2:**
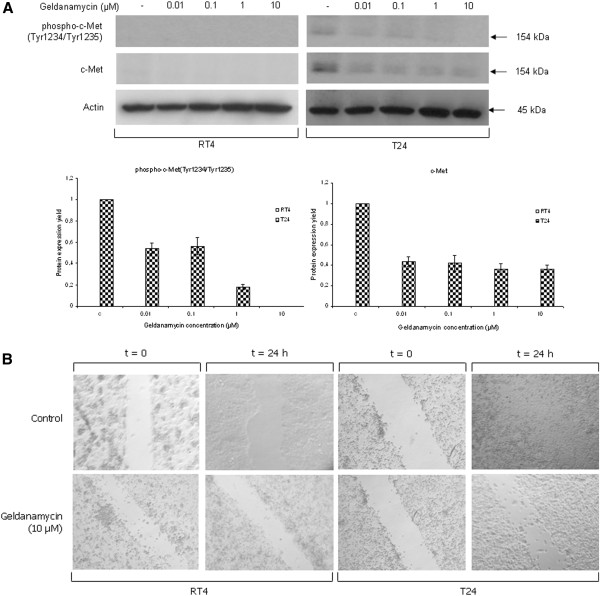
**Geldanamycin-induced cytotoxicity.** MTT toxicity assays were performed in RT4 and T24 cells after 24- (left) and 48-hour (right) treatment with increasing concentrations of geldanamycin. All assays were carried out three times. Standard deviation values are depicted as error bars on top of each value, while asterisks denote statistical significance of the observed averaged differences. *P* < 0.05 .

To study the biological effect of geldanamycin on bladder cancer cell survival, we performed MTT assays on RT4 and T24 cells, after their exposure to 0.01, 0.1, 1 and 10 μM of the drug for 24 and 48 hours. Both cell lines exhibited a dose-dependent reduction of cell viability, as illustrated in Figure 2. Survival levels at the highest concentration of geldanamycin (10 μM) were measured as 71 and 61% for the 24-hour treatment of RT4 and T24 cells, respectively. T24 cells presented with higher sensitivity to the cytotoxic activity of geldanamycin after 24 hours of treatment, compared to RT4 cells, therefore revealing the likely targeted capacity of the drug on cells with comparatively higher malignancy grade. Cell viability showed a stronger decrease for both cell lines after 48 (compared to 24) hours of geldanamycin exposure, as cell survival proved not to exceed the 62% ratio for differentiated RT4 cells and the 46% one for malignant T24 cells at the drug dose of 10 μΜ. Cells measured alive at 24 hours, albeit already committed to apoptosis, appeared to die after 48 hours of drug treatment. Hence, the percentage of cell survival is significantly reduced in both bladder cancer cell lines, with T24 cells being comparatively more vulnerable to the cytotoxic potency of gelanamycin. Activation of programmed cell death is the prevailing response of cells against chemotherapeutic agents. Survival rates obtained in this study directly reflect the cytotoxic and likely apoptotic (see below) function of geldanamycin-induced Hsp90 inhibition in RT4 and T24 cells, following a cell line-specific mode of action. T24 cells proved to respond better to the treatment, demonstrating comparatively lower resistance to the drug and therefore indicating geldanamycin’s targeted cytotoxic efficacy against highly malignant urothelial cells.

### Geldanamycin induces activation of cell death programs in bladder cancer cells

**Figure 3:**
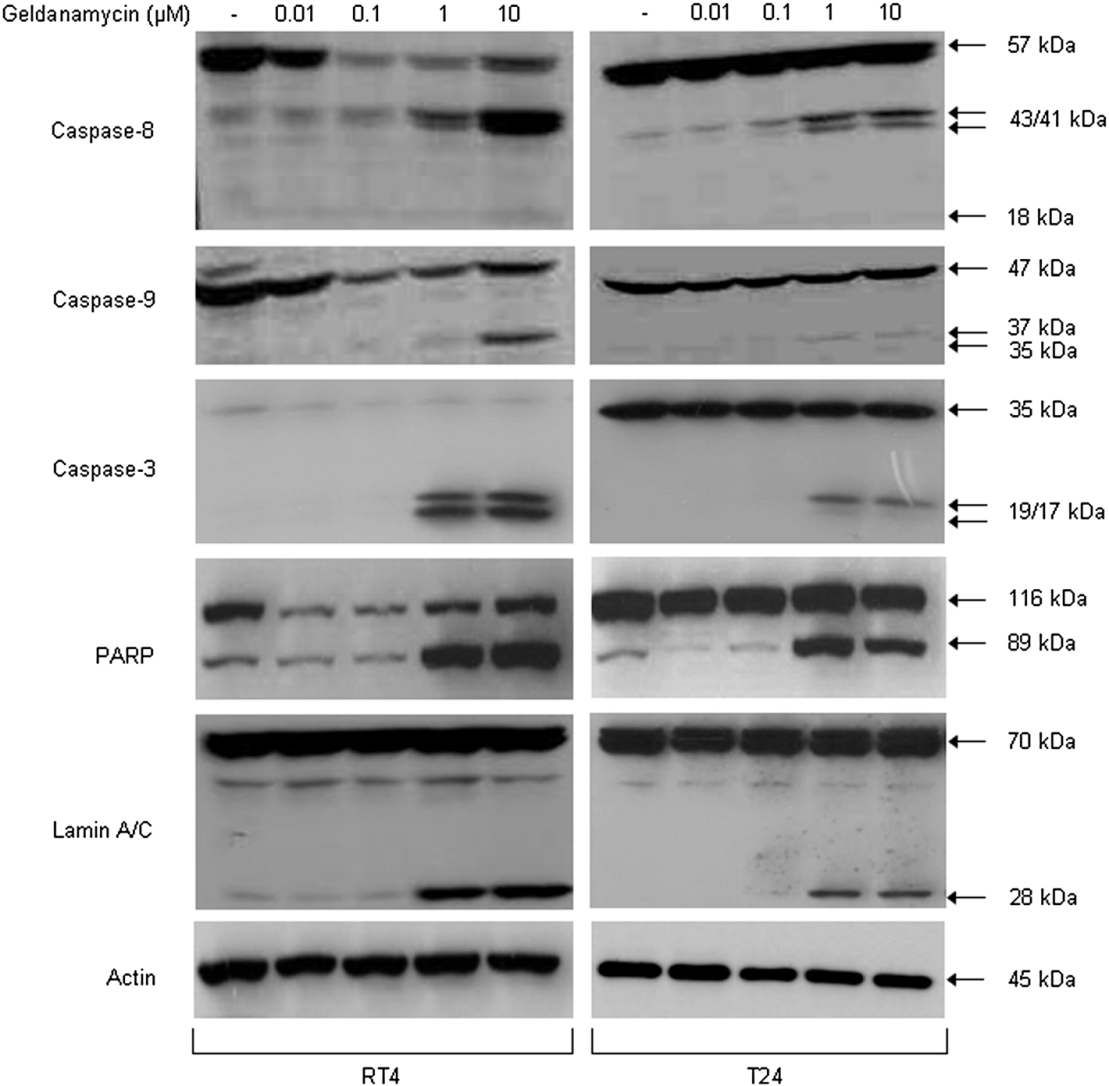
**Caspase-dependent activation of programmed cell death.** Expression and proteolytic processing patterns of apoptosis-related critical proteins in response to 24 hours of geldanamycin treatment in RT4 and T24 bladder cancer cells. Western blottings of members of the caspase signaling cascade (caspase-8, -9 and −3) and the caspase repertoire substrates PARP and Lamin A/C are shown. Actin has been used as protein of reference. Data are obtained from three different experimental trials, one of which is presented here.

Geldanamycin-induced reduction of cell survival was further investigated through a Western blotting approach and it proved to be strongly associated with proteolytic cleavage and activation of critical members of the caspase family, as shown in Figure 3. The downregulation profiles of precursor caspase protein expression levels, specifically evinced in RT4 cells, together with dose-dependent and cell line-specific increase of proteolytic cleavage product levels of the two initiator caspases (caspase-8 and caspase-9) and the executioner caspase-3, clearly demonstrate the ability of geldanamycin to induce apoptotic cell death via caspase-cascade activation in the bladder cancer cell lines examined. Apoptotic cell death was further certified by the detection of proteolytic processing of the caspase-repertoire substrates PARP and Lamin A/C upon administration of relatively high concentrations of geldanamycin (1 and 10 μΜ). The drug-induced cleaved fragments of PARP (89 kDa) and Lamin A/C (28 kDa) are characteristic markers of caspase-dependent apoptotic death, thus indicating the ability of geldanamycin to drive bladder cancer cells to apoptosis. However, it proved that T24 are more resistant to drug-induced apoptosis than RT4 cells, as clearly documented by the comparison of the obtained proteolytic profiles of the apoptotic regulators examined. The inconsistency between cell survival rates and caspase repertoire activation in drug-exposed T24 (compared to RT4) bladder cancer cells led us to examine the presumable implication of alternative and caspase-independent cell death programs that could tightly regulate the strong reduction of cell proliferation and survival in the T24 cell line in response to geldanamycin administration.

**Figure 4:**
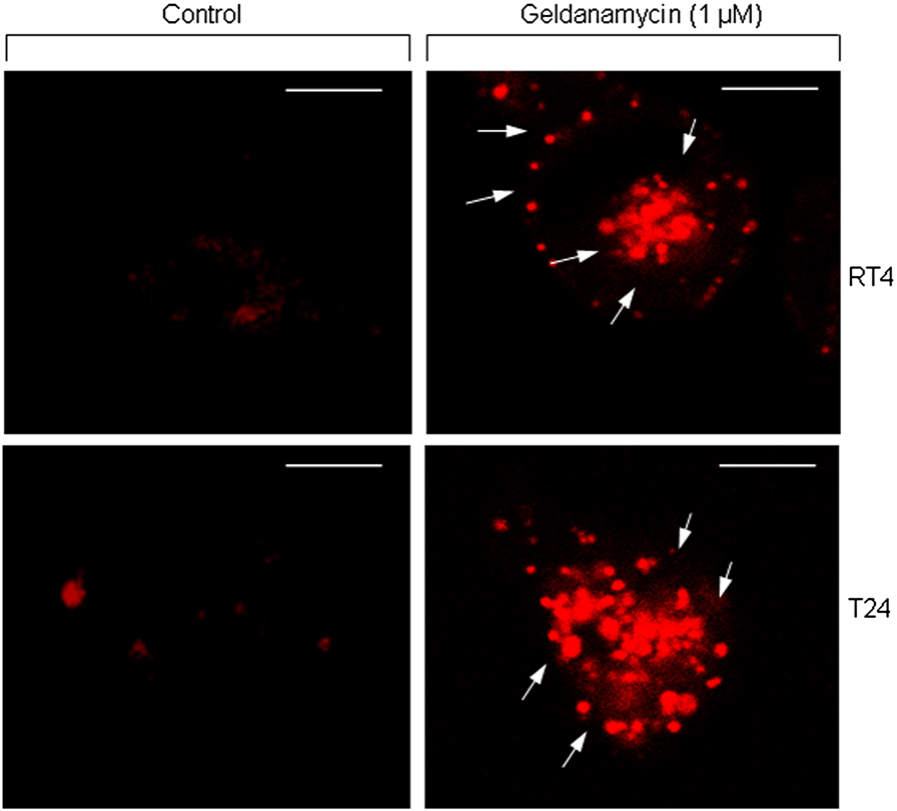
**Autophagy-mediated programmed cell death.** Characteristic images of RT4 and T24 bladder cancer cell activation of autophagy in response to 1 μM of geldanamycin treatment (scale bars: 10 μm). Cells were stained with Lysotracker Red that specifically recognizes active lysosomes (white arrows), therefore allowing the detection of an autophagic cell death program. Images were taken under a Nikon EZ-C1 confocal microscope. All cell staining experiments were conducted three times, while a representative image collection is shown here.

Figure 4 illustrates that RT4 and T24 cells can demonstrate significant levels of lysosomal activity upon 24-hour geldanamycin treatment, as clearly evinced by their incubation with an autophagy-specific cell dye (Lysotracker Red) and the suitable application of confocal microscopy technology. Therefore, it seems that lysosome-mediated autophagy critically contributes to the significant reduction of RT4 and T24 cell survival in response to Hsp90 inhibition. Although apoptosis is considered the dominant mechanism of programmed cell death upon drug administration [20,23], autophagy seems to come into play rather frequently when certain type of stress is applied [24,25]. As documented here, Hsp90 inhibition proves to awake the internal programmed cell death machineries, resulting in the engagement of distinct death cascades that likely operate in a cell line-specific fashion. This mainly occurs due to geldanamycin-induced caspase-3 activation, and its cognate substrates fragmentation, as well as lysosome-orchestrated autophagic cleavage of critical cellular proteins or protein complexes. Conclusively, we have established the simultaneous activation of apoptosis and autophagy that proved to decisively contribute to (bladder cancer) cell line-specific cytotoxicity of geldanamycin, unveiling drug’s strong efficacy to successfully eradicate urothelial cells of high malignancy.

### Hsp90 impairment due to exposure of human urinary bladder cancer cells to geldanamycin

**Figure 5:**
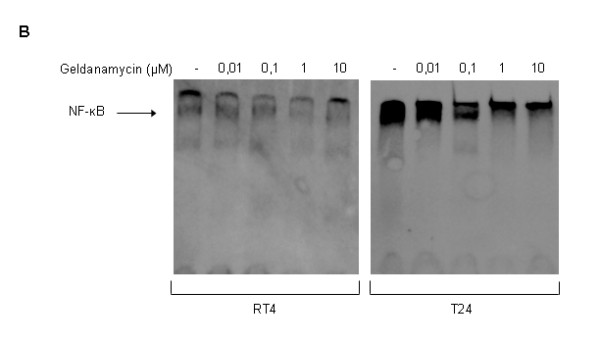
**Hsp90 harm after exposure of bladder cancer cells to geldanamycin.** Western blottings (upper left panel) of critical members of the eukaryotic chaperosome (Hsp90 and Hsp70) after 24-hour geldanamycin administration in RT4 and T24 bladder cancer cells. A cell line-specific Hsp90 proteolytic processing is observed, as documented by the production of a ~65 kDa Hsp90-like protein fragment. Detection of *Hsp90α* and *Hsp90β* mRNA levels via sqRT-PCR (lower left panel), proving the absence of Hsp90 transcriptional regulation in response to the drug. Right panel: protein (top and middle) and RNA transcript (bottom) densitometric quantification bars, denoting the drug-induced alterations of Hsp90/Hsp70/α-Tubulin and *Hsp90α*/*Hsp90β* expression levels compared to control conditions, using Actin (upper left panel) and *GAPDH* (lower left panel) as protein and gene of reference, respectively. Western blotting and sqRT-PCR experiments were executed three times, with a respective characteristic image collection presented here. Standard deviation values are depicted as error bars on top of each value.

Next, we studied the effect of geldanamycin on the structural and functional integrity of its molecular target; the Hsp90 chaperone. As shown in Figure 5 (upper image and respective graph), RT4 cells display a dose-dependent downregulation of Hsp90 protein levels up to the concentration of 0.1 μΜ. This pattern is disrupted by a following increase of chaperone’s expression levels that occurs under the actions of the higher concentrations (1 and 10 μM) of the drug. The complete restoration of total Hsp90 protein levels was accompanied by the production of a proteolytically cleaved Hsp90 fragment (~65 kDa) at the highest drug dose. This pattern was not detected in the malignant cell line T24, which displayed a mild and dose-dependent downregulation of Hsp90 levels in response to geldanamycin treatment. This intriguing finding has been reported by our research group in a recent study that revealed a cell line-specific proteolytic cleavage of Hsp90 as a response to 17-AAG, another Hsp90 inhibitor [20]. However, the experimental data presented herein indicate for a distinct and non-described, so far, inhibitory activity of geldanamycin on Hsp90, which is deployed by its ability to critically reduce Hsp90 cellular contents upon certain stress levels, specifically in an environment of bladder cancer cells of low malignancy grade. Specific conformations of the chaperone have been implicated in the sensitivity of cancer cells to Hsp90 inhibitors, compared to normal cells that seem to be less susceptible to these types of drugs [26]. In accordance, the prototype compound of the benzoquinone ansamycin group of natural antibiotics, geldanamycin, proves to exert strong impairment activities on the molecular chaperone Hsp90, therefore allowing the therapeutic exploitation of its high binding affinity to predominantly cancerous chaperone complexes.

The protein levels of the co-chaperone Hsp70 exhibited a dose-dependent increase in both drug-treated cell lines, while the production of an additional proteolytically cleaved product at the -mainly- higher doses of the drug (1 and 10 μM for RT4 cells; 0.1, 1 and 10 μM for T24 cells) was also observed (Figure 5 {upper image and respective graph}). Interestingly, the molecular weight of Hsp70 proteolytic fragment was calculated at approximately 65 kDa, similar to the one measured for Hsp90 cleaved product. Hsp90 and Hsp70 fragmentation has been previously reported in response to diverse types of stress [20,27] and seems to correlate well with Granzyme-B-mediated proteolytic processing of the chaperosome. The cell line-specific products of ~65 kDa could likely act as putative dominant negative components, able to induce a functional amputation of each molecular chaperone, respectively. Geldanamycin-mediated inhibition of Hsp90 also leads to similar -to Hsp90- protein expression profiles of molecules that are in direct structural and functional association with the molecular chaperone, such as the cytoskeleton component α-tubulin (Figure 5 {upper image and respective graph}). In order to mechanistically dissect the intriguing and fluctuation-type pattern of Hsp90 protein expression in response to increasing concentrations of geldanamycin, we, next, examined the transcriptional expression profiles of *Hsp90α* and *Ηsp90β* cognate genes upon geldanamycin administration. *Hsp90* (*α* and *β*) mRNA transcript levels proved to remain unaffected in both drug-exposed RT4 and T24 cells (Figure 5 {lower image and respective graphs}), thus excluding any type of transcriptional regulation mechanism in the geldanamycin-induced impairment of Hsp90 structural and functional integrity, in a tumorigenic urothelial environment.

### Exposure to geldanamycin leads to disruption of cell signaling activity

**Figure 6:**
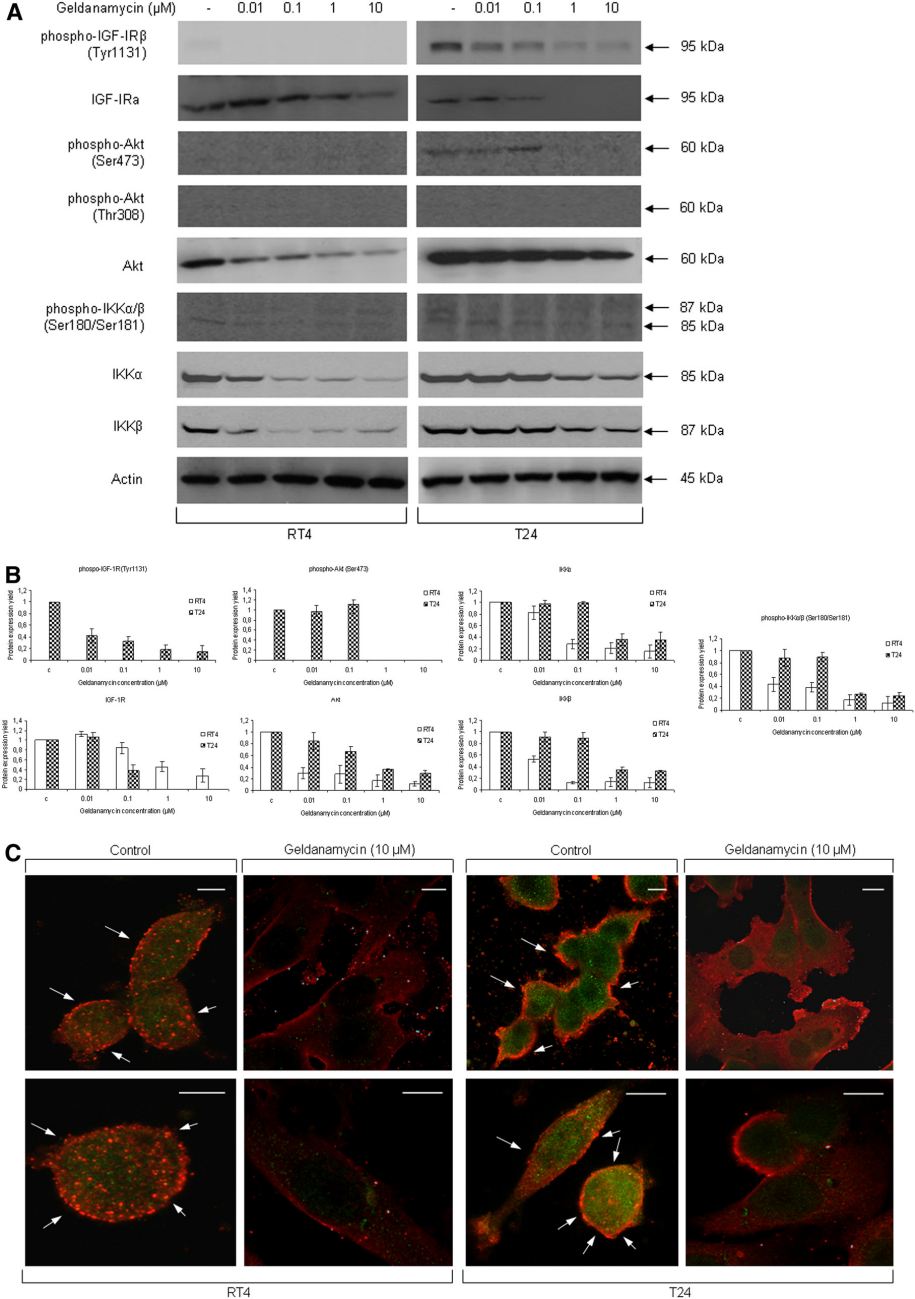
**Oncogenic signal transduction downregulation (I).** Geldanamycin-induced Hsp90 inhibition results in functional impairment of Akt-dependent signaling in human urinary bladder cancer cells. (**A**) Western blotting experiments reveal drastic downregulation of Akt signaling activity, evolving through the IGF-IR/Akt/IKK signal transduction axis, as evidenced by the eradication of both total and constitutively phosphorylated protein forms of all examined Hsp90 protein clients (IGF-IR, Akt, IKKα and IKKβ), in RT4 and T24 bladder cancer cell lines. Actin was used as protein of reference. All Western blottings were carried out three times, with a representative image collection shown here. (**B**) Protein densitometric quantification bars, denoting the drug-induced expression level alterations of the IGF-IR/Akt/IKK signaling axis components, and their respective phosphorylated forms, compared to control conditions, using Actin (A) as protein of reference. Standard deviation values are depicted as error bars on top of each value. (**C**) Immunofluorescence images illustrating the cellular localization of Hsp90 (red color) and Akt kinase (green color) in RT4 and T24 bladder cancer cells, in the presence or absence of geldanamycin. Upon drug administration (10 μΜ), a pronounced depletion of cytoplasmic Akt protein is detected, in contrast to what is observed under control conditions. The bright orange color (white arrows) indicates the cellular areas where Akt and Hsp90 are likely co-localized (associated), in the absence of drug conditions. Images were taken under a Nikon EZ-C1 confocal microscope. All immunofluorescence experiments were conducted three times, while a characteristic image collection is presented here (scale bars: 10 μm).

Structural and functional inhibition of Hp90 due to geldanamycin administration leads to severe downregulation of critical protein clients of the molecular chaperone in human urinary bladder cancer cells, as shown in Figure 6A. In response to increasing doses of geldanamycin, essential protein components of the IGF-IR/Akt/NF-κB signaling axis displayed prominent dose-dependent reduction of their expression levels. Total protein levels of IGF-I receptor (IGF-IR) presented with significant dose-dependent decrease in both bladder cancer cell lines, with T24 cells being more vulnerable to the drug. Interestingly, even though the phosphorylated IGF-IR form in T24 cells was similarly downregulated in a dose-dependent manner, RT4 cells showed no phosphorylation of the receptor, likely unmasking their low malignancy grade character (Figure 6A {image} and 6B {respective graphs}). A key molecule in many signaling pathways and prominent member of the Hsp90 clientele is the Akt serine/threonine kinase. Upon administration of geldanamycin, Akt total protein levels presented with a dose-dependent and cell line-specific pattern of strong downregulation, with RT4 cells exhibiting a more notable decrease, resulting in a severe eradication of the Akt kinase at the highest drug concentration (10 μM). T24 cells proved to contain a constitutively phosphorylated Akt kinase form (on a critical serine residue at position 473), whose expression levels appeared to follow a dose-dependent and strong downregulation in response to geldanamycin administration. In contrast, and according to their low malignancy grade, RT4 cells did not show any basal phosphorylation of the Akt kinase (Figure 6A {image} and 6B {respective graphs}). The drug-induced severe degradation of Akt protein was further investigated by immunofluorescence experiments. As shown in Figure 6C, Akt (green color) presented with a prominent reduction of the number of “foci” that reflected protein’s cellular topology, upon 24-hour drug administration (10 μΜ) in RT4 and T24 bladder cancer cells. Geldanamycin-induced Akt degradation proved to also deplete the Hsp90 chaperosome (red color), with a putative physical interaction between Hsp90 and Akt proteins being primarily revealed under control conditions (absence of drug), where the chaperone and its kinase client seemed to share common subcellular localization, as it was mainly demonstrated by the bright orange colored-pattern (white arrows).

Geldanamycin-induced Akt downregulation was accompanied by degradation of its direct downstream targets and *bona fide* Hsp90 protein clients IKKα and IKKβ. Both IKK serine/threonine kinases presented with a dose-dependent pattern of total protein downregulation in both drug-exposed cell lines, with RT4 cells exhibiting a more severe reduction of expression levels, under the effect of increasing drug concentrations. Constitutively active ΙΚΚα and ΙΚΚβ kinases (phosphorylation on the critical serine residue 180 and 181, respectively) were detected in both RT4 and T24 cell lines and proved to follow a moderate dose-dependent reduction of signaling capacity in response to geldanamycin administration (Figure 6A {image} and 6B {respective graphs}). Conclusively, it seems that geldanamycin-mediated inhibition of Hsp90 leads to multiple structural truncations and functional amputations of critical molecules that constitute and modulate the IGF-IR/Akt/IKK signaling axis, thus rendering its signal transduction power severely compromised.

**Figure 7:**
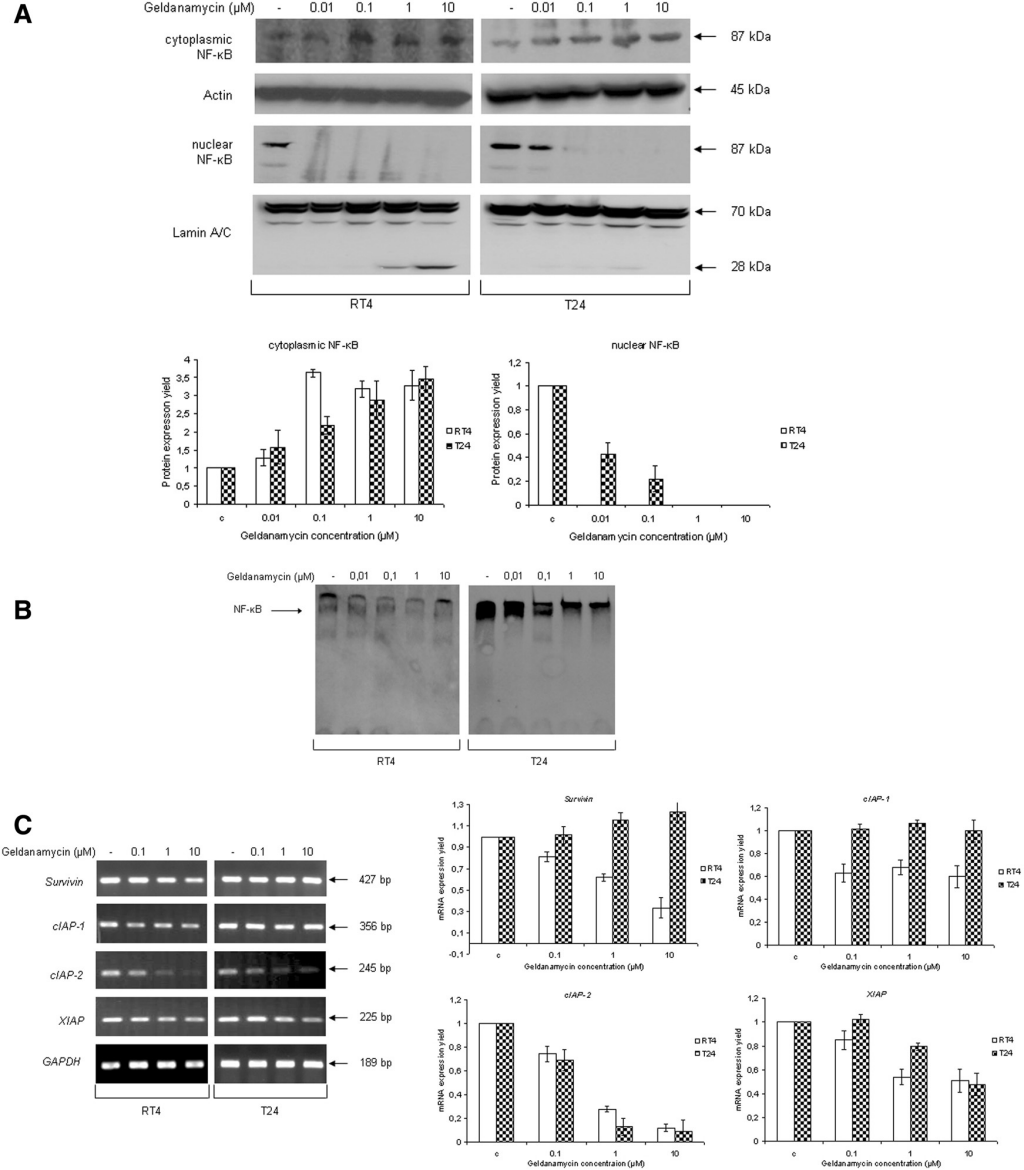
**NF-κΒ inactivation after exposure of human bladder cancer cells to geldanamycin.** NF-κB transcriptional capacity is diminished upon 24-hour geldanamycin administration in RT4 and T24 bladder cancer cells. (**A**) Upper panel: Western blottings displaying NF-κB protein expression levels and compartmentalization responses to drug treatment in both cytoplasmic and nuclear protein extracts of RT4 and T24 cells. Protein sample quantification of cytoplasmic and nuclear extracts was evaluated using Actin and Lamin A/C as respective (compartment-specific) reference markers, while the absence of detectable NF-κB in nuclear extracts derived from drug-treated cells ensured samples’ purity. Lower panel: cytoplasmic and nuclear NF-κB densitometric quantification bars, denoting its drug-induced compartmentalization change compared to control conditions, using Actin and Lamin A/C as respective proteins of reference. Standard deviation values are depicted as error bars on top of each value. Experiments were carried out three times, while characteristic Western blotting results are presented here. (**B**) Electrophoretic mobility shift assay (EMSA) conducted on RT4 and T24 nuclear protein extracts. Binding assays were performed using NF-κB-specific and 5´ -biotin labeled, double-stranded (annealed), oligonucleotides. EMSA experiments were repeated three times, one of which is shown here. (**C**) Left panel: transcriptional expression profiles of four critical anti-apoptotic NF-κB target genes (*Survivin*, *cIAP-1*, *cIAP-2* and *XIAP*), in response to 24-hour geldanamycin administration, in RT4 and T24 bladder cancer cells. Right panel: RNA transcript densitometric quantification bars, denoting the drug-induced expression level alterations of the NF-κB target genes examined, compared to control conditions, using *GAPDH* (left panel) as gene of reference. All sqRT-PCR reactions were carried out three times, while a characteristic assembled profiling is presented here. Standard deviation values are depicted as error bars on top of each value.

In order to further dissect this “pharmaco-signaling” pathway, we examined the effect of geldanamycin-induced Hsp90 inhibition on the endmost downstream target of IGF-IR/Akt/IKK signaling cascade, the NF-κB transcription factor. As shown in Figure 7A, geldanamycin forces the relocation of NF-κB from cell nucleus to cytoplasm, thus restraining its -constitutive- transcriptional activity, mainly due to its drug-mediated sequestration into the cytoplasm. As evidenced by Western blotting, cytoplasmic protein extracts displayed a dose-dependent increase of NF-κB expression levels in both drug-treated cell lines, while nuclear extracts presented with a complete depletion of the transcription factor under the actions of increasing doses of the drug, with T24 being relatively more resistant than RT4 cells (Figure 7A {image and respective graphs}). To reinforce the biological significance of geldanamycin-induced NF-κB inactivation observed, we performed EMSA assays, using RT4 and T24 nuclear extracts, and found that the sequestration of NF-κB into the cytoplasm was tightly associated with its dose-specific inability to recognize and bind onto its 5´ -biotin labeled oligonucleotide target sequence, in response to geldanamycin (Figure 7B). Next, we also attempted to rather more directly assess the drug-induced damage of NF-κB transcriptional activity, by examining the mRNA expression profiles -through sqRT-PCR- of several *bona fide* NF-κB target genes (i.e. *Survivin*, *cIAP-1*, *cIAP-2* and *XIAP*) after exposure of RT4 and T24 cells to geldanamycin. As presented in Figure 7C (image and respective graphs), all four genes displayed cell line-specific and dose-dependent downregulation profiles upon drug exposure, with *cIAP-2* showing the strongest effect. The transcriptional repression of this group of anti-apoptotic genes strongly reflects the severe disruption of critical signaling pathways due to geldanamycin administration, therefore contributing to the anti-neoplastic and anti-proliferative capacity of Hsp90 inhibition on bladder cancer cells. IGF-IR/Akt/NF-κB signaling axis deregulation plays an essential role in cancer cell survival, tumor proliferation and migration [20,28,29]. Conclusively, it seems that geldanamycin administration drastically compromises intracellular signaling of neoplastic urothelial cells due to targeted kinase degradation, ultimately resulting in the transcriptional repression of several anti-apoptotic genes upon NF-κΒ sequestration into the cytoplasm, as previously suggested for a “chemoradiotherapy” type of applied stress [28]. This multi-combinatorial “attack” of the drug to cancer cell signaling and transcriptional networks directly reflects the strong therapeutic potential of Hsp90 inhibition in human bladder cancer and unmasks geldanamycin’s anti-tumorigenic properties.

**Figure 8:**
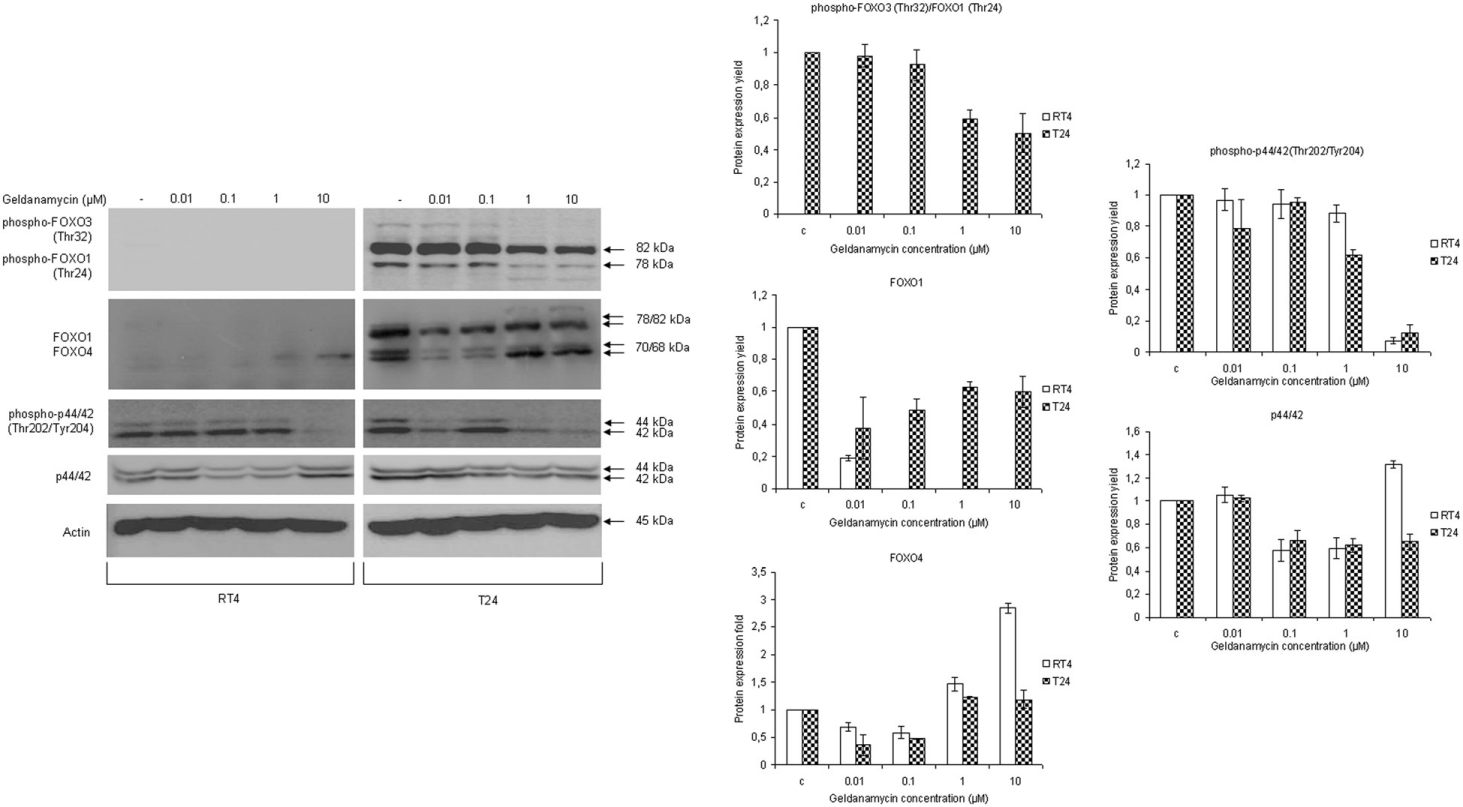
**Oncogenic signal transduction downregulation (II).** Left panel: Western blotting experiments presenting protein expression levels of total and constitutively phosphorylated FOXO transcription factors and p44/42 MAP kinases, in RT4 and T24 bladder cancer cells, upon geldanamycin administration for 24 hours. Right panel: protein densitometric quantification bars, denoting the drug-induced expression level alterations of the FOXO and MAPK family members examined, and their respective phosphorylated forms, compared to control conditions, using Actin (left panel) as protein of reference. All experiments were repeated three times, with a typically obtained image collection shown here. Standard deviation values are depicted as error bars on top of each value.

In an effort to further assess the combinatorial action of Hsp90 inhibition on critical signal transduction cascades with known cross-talk functions between each other, we studied the effect of geldanamycin-induced Hsp90 inhibition on the Akt downstream signaling targets FOXO transcription factors and p44/42 MAP (Erk1/2) kinases, which are tightly associated with the regulation of cell proliferation and survival. As shown in Figure 8 (image and respective graphs), in contrast to RT4, T24 cells were characterized by constitutively phosphorylated FOXO1 and FOXO3 proteins that displayed dose-dependent downregulation profiles in response to geldanamycin. Therefore, a dephosphorylation-mediated nuclear sequestration of FOXO factors seems to represent a predominant response to the drug that results in the following trans-activation of certain apoptotic target genes, therefore reinforcing the generation of an apoptotic phenotype. Next, we examined the effect of geldanamycin-induced Hsp90 inhibition on the MAP kinase signaling cascade, through the detection of total and phosphorylated protein levels of p44 and p42 serine/threonine MAP kinases, using the Western blotting technology. As evinced in Figure 8 (image and respective graph), total protein levels of p44/42 kinases in RT4 cells presented with a drug response pattern similar to the ones described above for Hsp90, Cdk4 and α-tubulin proteins. In T24 cells, p44/42 total proteins were moderately downregulated in a dose-dependent manner in response to geldanamycin. Moreover, we analyzed the signaling capacity of p44/42 kinases through detection of the active (phosphorylated on the critical residue threonine 202 and tyrosine 204, respectively) proteins in the presence or absence of the drug. Figure 8 (image and respective graph) reveals that both RT4 and T24 cells present with constitutively active (phosphorylated) forms of p44/42 proteins, which undergo dose-specific inactivation after exposure to geldanamycin, with T24 cells being comparatively more sensitive to the higher drug doses. MAP kinase signaling attenuation and damage likely result in downregulation and deactivation of several downstream targets critically implicated in cell survival and proliferation, thus rendering bladder cancer cells susceptible to the cytotoxic power of geldanamycin. Functional deregulation of FOXO- and p44/42-dependent signaling pathways is critical to tumor survival and growth, while their inhibition is known to contribute to the production of an apoptotic phenotype [30]. It seems that geldanamycin in human urinary bladder cancer cells causes a strong suppression of pivotal tumorigenic signaling networks, therefore inducing activation of apoptosis and contributing to obliteration of malignant characteristics.

### Geldanamycin administration results in impaired bladder cancer cell motility

**Figure 9:**
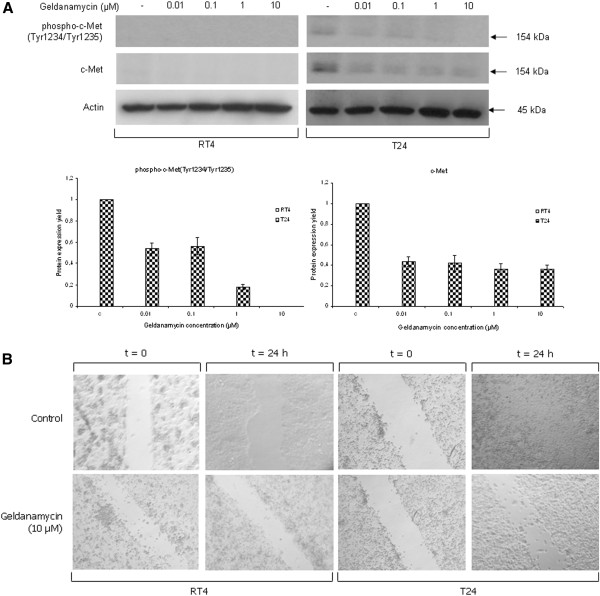
**Impairment of bladder cancer cell motility and invasion mechanisms.** (**A**) Top panel: total and phosphorylated protein expression levels of the hepatocyte growth factor receptor (c-Met), upon 24-hour incubation of RT4 and T24 bladder cancer cells with increasing doses of geldanamycin, as shown by Western blotting. Bottom panel: protein densitometric quantification bars, denoting the drug-induced downregulation of total and phosphorylated c-Met expression levels, compared to control conditions, using Actin (top panel) as protein of reference. All Western blottings were performed three times, with a characteristic image collection presented here. Standard deviation values are depicted as error bars on top of each value. (**B**) Scratch-wound assays were carried out in RT4 and T24 bladder cancer cells under control conditions or treatment with 10 μM of geldanamycin for 24 hours. Observations were made under an inverted microscope (Carl Zeiss Axiovert 25) and pictures were taken at 20x magnification. All assays were repeated three times, while a representative image collection is shown here.

Cancer cell motility is one of the “hallmark traits” of the neoplastic phenotype and plays an important role in the process of tumor cell invasion and tissue infiltration. c-Met, also known as the hepatocyte growth factor (HGF) receptor, is a key signaling component in tumor proliferation, cancer cell motility and invasion [31]. In this context, we studied the effect of geldanamycin on c-Met signaling and cancer cell motility via Western blotting and scratch-wound assay, respectively. As shown in Figure 9A (image and respective graphs), constitutively phosphorylated (on critical tyrosine residues at positions 1234 and 1235) and total c-Met protein levels were detected only in the aggressive and highly malignant T24 cells, and not in the low malignancy grade RT4 cells. Both -constitutively- active and total c-Met protein contents in T24 cells proved to follow dose-dependent downregulation profiles in response to geldanamycin administration, thus indicating the suppressive effect of Hsp90 inhibition on bladder cancer cell motility and invasion dynamics.

To further confirm the ability of drug-induced signaling decay to abrogate cancer invasion, we attempted to morphologically visualize the confinement of malignant cell motility through scratch-wound assays. As presented in Figure 9B, RT4 and T24 cells failed to successfully “heal” a -mechanically- created gap in their cell monolayer surface during a 24-hour period of geldanamycin (10 μM) exposure, whereas under control conditions (absence of the drug) the wound was completely “healed” (forming an uninterrupted monolayer) in T24 and weakly “fixed” in RT4 cells on the assay surface of Petri dish. The geldanamycin-induced c-Met signaling attenuation reported here accords with previous observations in different cellular environments [32], strongly evidencing the severe damage in cell proliferation and motility processes of urinary bladder cancer cells growing under the baneful actions of this naturally derived and prototype Hsp90 inhibitor, the geldanamycin.

## Conclusions

**Figure 10:**
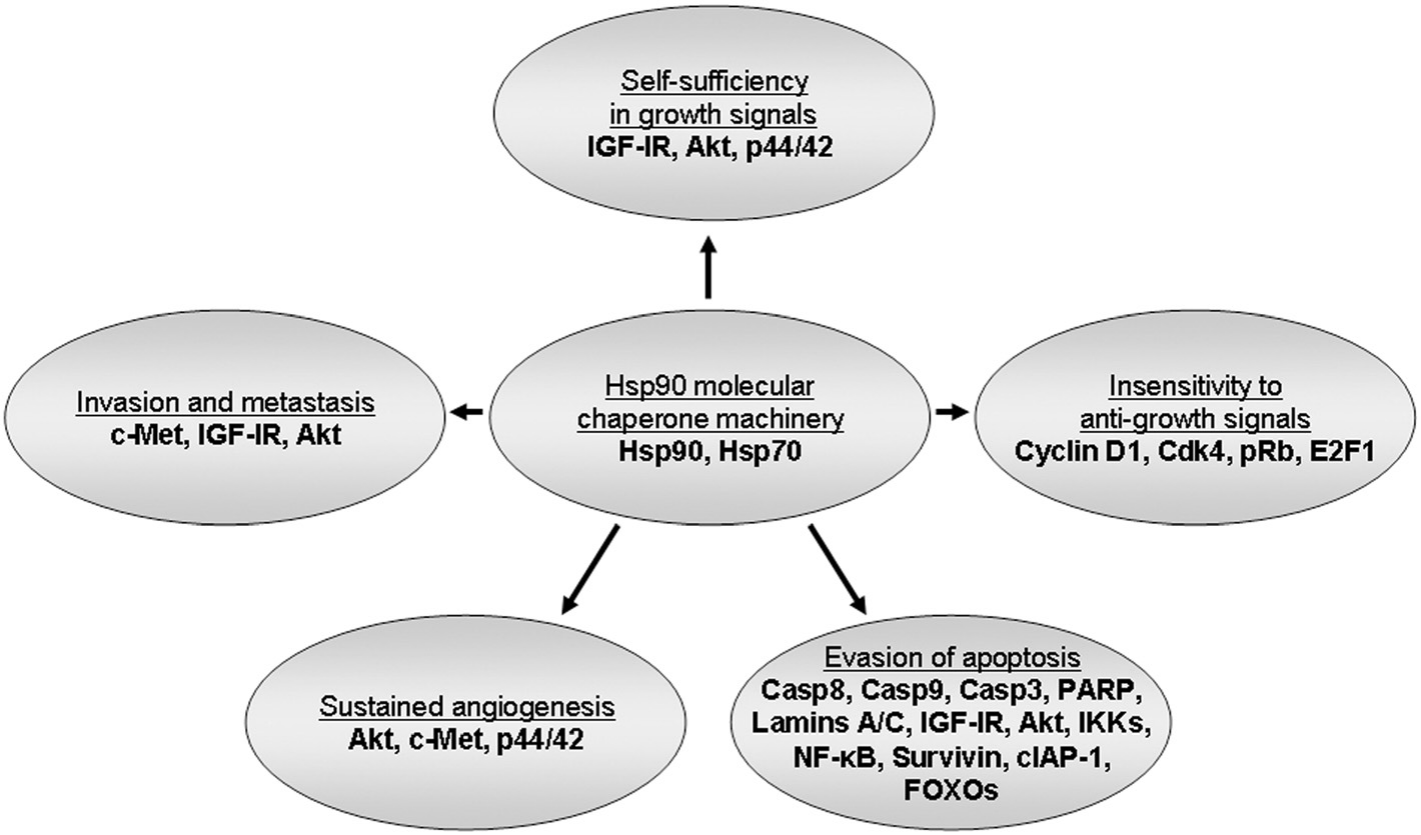
**Hsp90 inhibition eliminates the “hallmark traits” of cancer.** The data presented here clearly demonstrate that the geldanamycin-induced inhibition of Hsp90 results in the combinatorial and multi-step impairment of critical oncogenic pathways likely implicated in the “hallmark traits” of bladder cancer initiation and progression.

In this study, we have demonstrated the anti-neoplastic properties of the benzoquinone antibiotic geldanamycin in human urinary bladder cancer cell lines of different malignancy grade. The obtained results proved the dose-dependent and prominent elimination of “hallmark traits” of cancer in both RT4 and T24 cells upon geldanamycin-induced inhibition of Hsp90 molecular chaperone (Figure 10). Drug-mediated downregulation of multiple Hsp90 protein clients in bladder cancer cellular environments resulted in cell cycle arrest, disruption of tumorigenic signal transduction integrity, and impairment of cell motility and invasion mechanisms, whereas cell death programs in the form of apoptosis and autophagy were strongly activated in response to geldanamycin. Pre-clinical *in vivo* studies have reported side effects of systemic hepatotoxicity [33], hence limiting the “wide-spreading” utilization of the drug in human malignancies. However, orthotopic administration of -clinically relevant- low doses of geldanamycin into the affected bladder could prove an effective therapeutic approach, initially sensitizing tumors to chemoradiotherapy (CRT) protocols, not only by successfully deploying a combinatorial “attack” to multiple oncogenic pathways involved in “hallmark traits” of cancer, but by simultaneously circumventing hepatic toxicity of the drug.

## Materials and methods

### Drugs and reagents

Geldanamycin was obtained from InvivoGen (San Diego, California, USA). Antibodies against Hsp90α/β, Hsp70, Cdk4, pRb and E2F1 were supplied by Santa Cruz Biotechnology Inc. (California, USA), whereas the rest of the polyclonal and monoclonal antibodies used herein were purchased from Cell Signaling Technology Inc. (Hertfordshire, UK). Enhanced Chemilluminescence (ECL) Western blotting detection reagents were obtained from GE Healthcare Life Sciences (Buckinghamshire, UK). Oligonucleotide primers were synthesized by Operon (California, USA). All other chemicals were of analytical grade from Sigma-Aldrich (Missouri, USA), Fluka (Hannover, Germany) and AppliChem GmbH (Darmstadt, Germany).

### Cell lines and culture conditions

The present study was performed using as a basic biological system two human urinary bladder cancer cell lines, namely RT4 and T24, both originating from urothelial carcinomas. RT4 cells were derived from a grade I tumor and obtained from the European Collection of Animal Cell Cultures (Salisbury, UK), whereas T24 cells were derived from a grade III tumor and provided to us as a generous gift from Professor J. R. Masters (Prostate Cancer Research Centre, Institute of Urology, University College London, London, UK). All cell culture media and reagents were supplied by Biochrom AG (Berlin, Germany).

### Cell cycle analysis

Geldanamycin’s effect on bladder cancer cell cycle progression was analyzed via FACS approach as previously described [23]. Cells were stained with propidium iodide solution (50 μg/ml) containing 250 μg of DNase-free RNase A and subsequently analyzed with a Beckton Dickinson’s FACScalibur (California, USA) at 542 nm. Results were accordingly modified with the Modfit software program.

### Cell viability assay

Cytotoxicity of geldanamycin was monitored by the methylthiazole tetrazolium (MTT) assay as previously described [23]. Spectrophotometric absorbance was measured in an ELISA microtiter plate reader (Dynatech MR5000, Dynatech Laboratories, Virginia, USA) at 550 nm, using measurement at 630 nm as reference.

### Western immunoblotting

Whole cell protein extracts were prepared and appropriately resolved under denaturing conditions by SDS-Poly-Acrylamide Gel Electrophoresis. Nuclear and cytoplasmic protein extracts were obtained with the use of NE-PER extraction kit (Pierce, USA), while the subsequent immunodetection of NF-κB cellular compartmentalization was carried out using a suitable primary antibody. Membrane blocking, antibody incubation (diluted 1:1000 for all primary and 1:2000 for the two secondary antibodies used) and immunoreacting protein detection conditions were performed as previously described [23].

### sqRT-PCR analysis

Total RNA from both control and drug-treated cells was extracted as previously described [23]. The respective gene names, DNA nucleotide sequences, annealing temperatures and number of reiterative PCR cycles for the utilized cDNA primers have been previously described [20]. Semi-quantitative (sq) PCR protocols of high stringency were appropriately applied, in order to reliably quantify the geldanamycin-induced differences of transcriptional expression profiles of the examined genes (Figures 1B, 5 and 7C). The produced PCR fragments, after the suitable application of sqRT-PCR technology, were resolved in 2-3% agarose gels according to standard procedures [20].

### Electrophoretic Mobility Shift Assay (EMSA)

Nuclear protein extracts from cells grown in the absence or presence of geldanamycin were examined for NF-κΒ-containing activity under non-denaturating conditions in Poly-Acrylamide Gel Electrophoresis. Annealed 5´ -biotin labelled oligonucleotide probes (sense: 5´ -*AGTTGAGGGGACTTTCCCAGGC*-3´ and antisense: 5´ -*GCCTGGGAAAGTCCCCTCAACT*-3´) were used in order to determine the binding capacity of NF-κB nuclear complex on its cognate DNA target sequence in EMSA reactions. Assays were carried out according to the Light-Shift Chemilluminescent EMSA kit instructions (Pierce, USA), whereas immunoreacting protein detection was performed as previously described [20]. Experiments were conducted three times.

### *In situ* autophagy detection

Lysosomal-mediated programmed cell death was studied via the autophagy-specific and cell-reacting dye Lysotracker Red (Life Technologies Corporation, USA), and subsequently visualized under a Nikon EZ-C1 confocal microscope (Nikon Instruments Inc., Tokyo, Japan). Images were appropriately processed with the support of Nikon EZ-C1 software program. Experiments were repeated three times.

### Immunofluorescence

Human urinary bladder cancer cells were seeded on poly-L-lysine coated slides (Thermo Fisher Scientific Inc., Minnesota, USA) and treated with a geldanamycin concentration of 10 μM for 24 h. Fixation, cell permeabilization and subsequent immunodetection were carried out as previously described [20]. Secondary antibodies used herein were the DyLight 488 (green) and DyLight 633 (red) (Thermo Fischer Scientific Inc., Minnesota, USA). Cells were observed under a Nikon EZ-C1 confocal microscope (Nikon Instruments Inc., Tokyo, Japan). Images taken were appropriately processed with the Nikon EZ-C1 software program. Immunofluorescence experiments were conducted three times.

### Scratch-wound assay

Human urinary bladder cancer cells (5x10^5^) were seeded on 100 mm diameter Petri dishes and allowed to grow for 24 h in the presence or absence of geldanamycin (10 μM). Scratch-wound assays were performed as previously described [20]. Cell images were taken under a Carl Zeiss Axiovert 25 inverted microscope (Thornwood, New York, USA), with the use of a Cannon Powershot G9 digital camera and a PS-Remote software program. Scratch-wound assays were repeated three times.

## Competing interests

The authors declare that they have no competing interests, whatsoever.

## Authors’ contributions

PKK carried out most of the experimental work and drafted the manuscript. DJS performed confocal microscopy, participated in the design of the study and helped to draft the manuscript. EGK assisted in the maintenance of cell line cultures and sqRT-PCR experiments. GEV conceived the study, designed the sqRT-PCR oligonucleotide primers, participated in the design and coordination of the whole study and helped to draft the manuscript. All authors read and approved the final manuscript.
